# A 60-year-old woman with asymptomatic total thoracic–abdominal aortic aneurysm

**DOI:** 10.1186/s13019-021-01600-0

**Published:** 2021-08-04

**Authors:** Jianying Deng, Wei Liu

**Affiliations:** 1 Department of Cardiovascular Surgery, Chongqing Kanghua Zhonglian Cardiovascular Hospital, 168# Haier Street, Jiangbei District, Chongqing, 400015 China; 2 Department of Cardiac Surgery, DeltaHealth Hospital, Shanghai, 201720 China

**Keywords:** Aortic aneurysm, Staged repair, Surgery

## Abstract

**Introduction:**

Total thoracic–abdominal aortic aneurysm is a rare disease in cardiovascular surgery, with high surgical risk and high mortality. Surgery is considered the most effective treatment for total aortic aneurysms.

**Case presentation:**

Our group admitted a 60-year-old female patients with asymptomatic complex total thoracic–abdominal aortic aneurysm, and successfully performed two-staged surgery, namely Bentall + Sun’s operation in the first-stage and thoracoabdominal aortic replacement in the second-stage. The results of the surgery were satisfactory.

**Conclusions:**

Patients with total thoracic–abdominal aortic aneurysm may not have typical clinical symptoms and require a careful and comprehensive physical examination and related auxiliary examinations by clinicians. Staged repair of total thoracic–abdominal aortic aneurysms is still a safe and effective treatment.

## Introduction

Total thoracic–abdominal aortic aneurysm refers to aortic aneurysm lesions that span the diaphragm and involve the thoracic and abdominal aorta. Involving important organs and arteries throughout the body, therefore, the thoracic and abdominal aorta should be repaired. In recent years, despite the vigorous development of endovascular treatment, surgical repair technology is still an important method for the successful treatment of total thoracic–abdominal aortic aneurysms.

## Case presentation

**Fig. 1:**
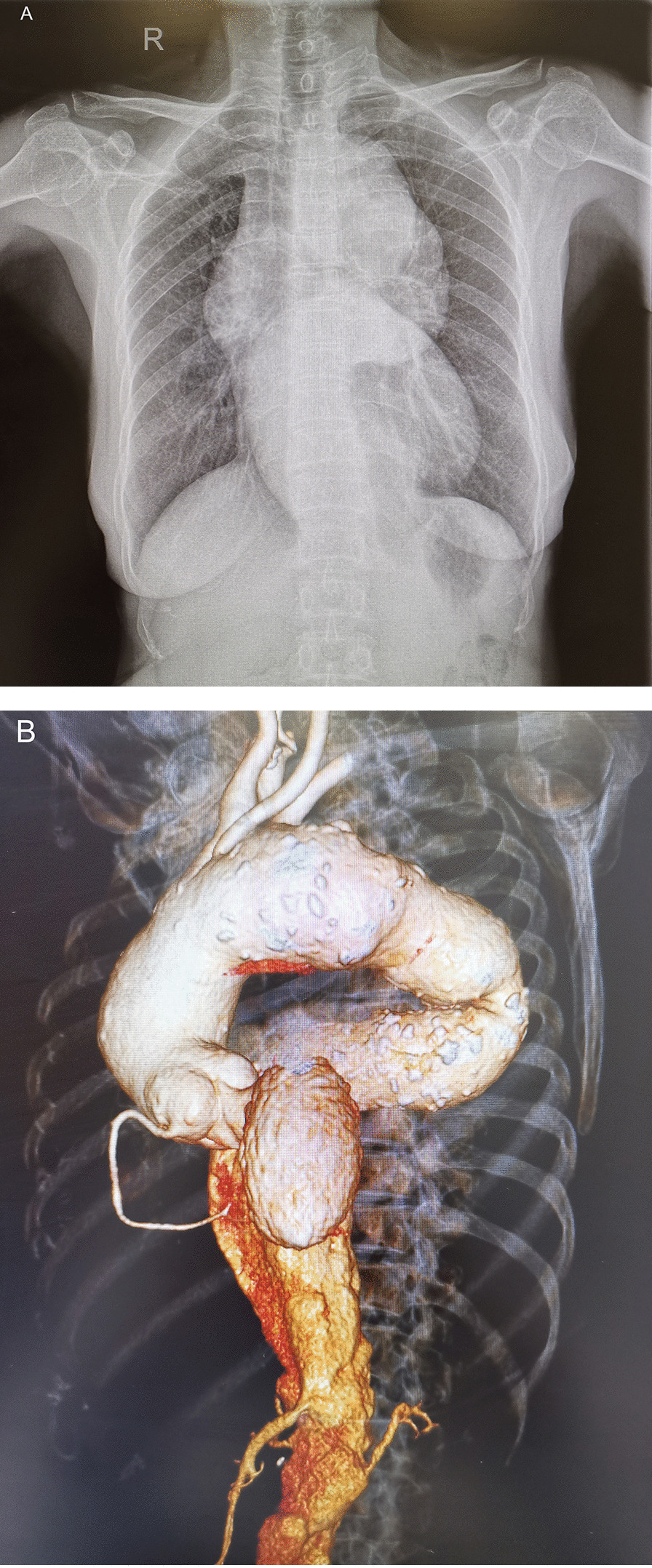
Chest radiograph showed a huge mass in the upper mediastinum (**A**); CTA showed a total aortic aneurysm, and the lesion involved the aortic root, ascending aorta, aortic arch, and the entire thoraco-abdominal aorta. The aneurysm is tortuous and deformed, mural thrombus can be seen in the aortic arch and abdominal aorta, multiple penetrating ulcers can be seen also in the thoracic and abdominal aorta, and the widest part of the aneurysm is 72 mm in diameter (**B**)

A 60-year-old female patient was admitted to our hospital with a complaint of complex aneurysm of the entire thoracic–abdominal aorta. Two month ago, the patient’s routine chest x-ray revealed a huge mass in the upper mediastinum (Fig. 1A), and further chest CT revealed an aneurysm of the total thoracic–abdominal aorta (Fig. 1B). For further surgical treatment, she was transferred to our hospital.

On admission, his vital signs were stable, no fever, no chest and abdominal pain. Blood pressure, 132/78 mmHg; heart rate, 80 beats/min; body temperature, 36.4℃; respiratory rate, 16 beats/min; arterial oxygen saturation on room air, 97%. The height is 162 cm, the weight is 65 kg, and the body mass index is 24.8 kg/m^2^. The patient's eye vision is normal. There was no abnormal growth in the bones of her limbs. She has a history of hypertension with well controlled. She denies that there are genetic diseases such as Marfan syndrome in her family, and she denies her personal history of drug abuse and promiscuity.

The laboratory test results are as follows: total cholesterol 5.8 mmol/L, triglyceride 2.09 mmol/L, uric acid 426umol/L. Transthoracic echocardiography showed that the patient’s aortic sinus was widened (maximum diameter 50 mm), and the aortic valve had moderate to severe regurgitation. Aortic CTA indicates aortic aneurysm, lesions involving the ascending aorta, aortic arch, thoracic–abdominal aorta, aortic arch and abdominal aortic wall thrombus, multiple penetrating ulcers in the thoracic–abdominal aorta, 58 mm at the widest part of the ascending aorta, diameter of the aortic arch 59 mm, 72 mm at the widest part of the thoracic aorta, and 57 mm at the widest part of the subrenal abdominal aorta (Fig. 1B).

Given that the patient’s entire thoracic–abdominal aortic aneurysm is very complex and older, after thorough discussion and full communication with the patient and family members, our team believes that the risk of two-staged total thoracic–abdominal aortic replacement surgery is less than of the one-staged total thoracoabdominal aortic replacement. We finally decided to implement two-staged surgery, namely Bentall procedure and Sun’s operation in the first-stage and thoracoabdominal aortic replacement in the second-stage.

**Fig. 2:**
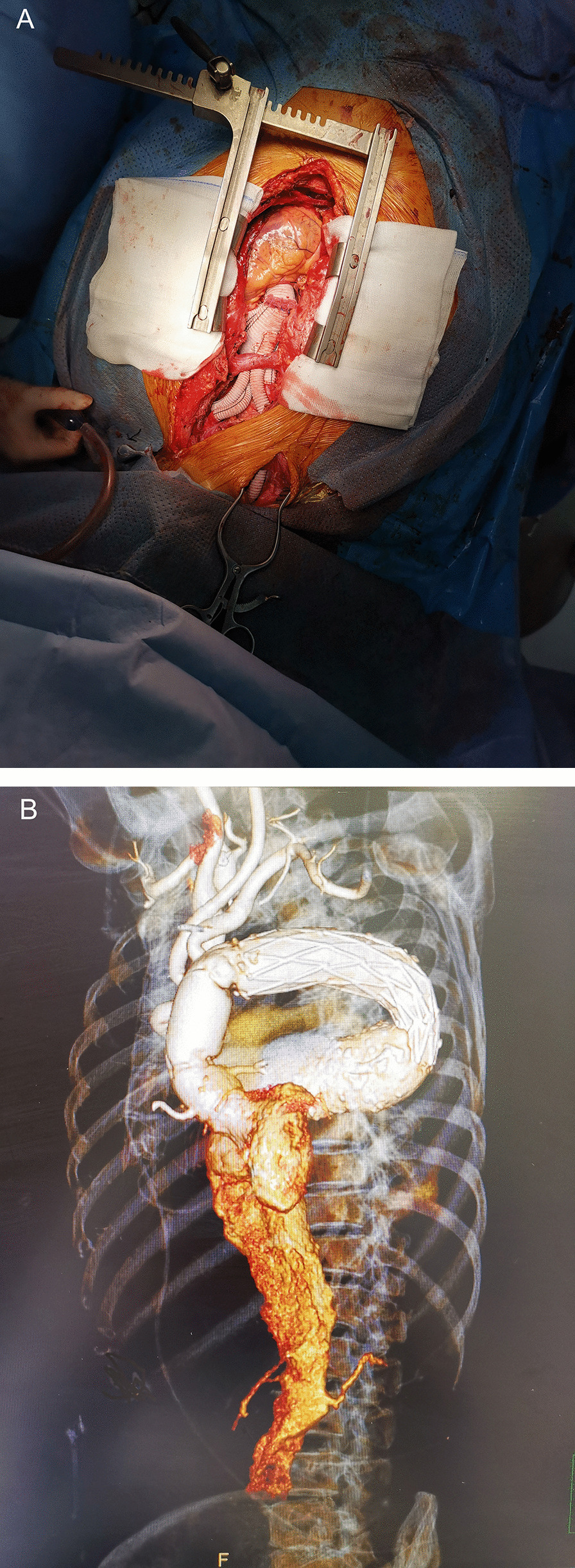
This patient successfully underwent the first-stage of Bentall and Sun’s procedure (**A**), and the postoperative CT (**B**) showed that the aortic root, ascending aorta, aortic arch, and proximal descending aorta were completely eliminated

During the first-stage of Bentall + Sun’s operation, we routinely use median sternotomy, right axillary artery and right atrium cannulation to establish cardiopulmonary bypass, and blow CO_2_ into the surgical field. After blocking the ascending aorta, the cardioplegia was perfused directly through the opening of the left and right coronary arteries. During the cooling process, the Bentall procedure of the aortic root was completed. When the nasopharyngeal temperature drops to 24–25 °C and the rectal temperature drops to 26–28 °C, the patient’s head was lowered by 20°–30°. The innominate artery, left common carotid artery and left subclavian artery were blocked respectively, and selective cerebral perfusion was performed, and the perfusion flow rate was reduced to 5 mL/kg min. During the operation, according to the monitoring of bilateral cerebral oxygen or the amount of blood returned to the left common carotid artery, decide whether to choose bilateral cerebral perfusion. Most patients with good collateral circulation in the basilar artery ring (Williams) can tolerate unilateral cerebral perfusion. After placing a suitable elephant trunk stent during the operation, the distal aortic anastomosis was quickly completed. After the left common carotid artery anastomosis is completed, the perfusion flow is gradually restored through one of the 4-branch artificial blood vessel, and bilateral cerebral perfusion and systemic perfusion are performed, and the temperature is slowly rewarmed. Then anastomose the left subclavian artery, the proximal end of the artificial blood vessel, and finally the innominate artery to complete the Bentall + Sun’s operation (Fig. 2A). After the first-stage of Bentall procedure and Sun’s operation was successfully performed, a re-examination of CTA showed that the lesions of aneurysm in the aortic root, ascending aorta, and aortic arch were well treated (Fig. 2B).

**Fig. 3:**
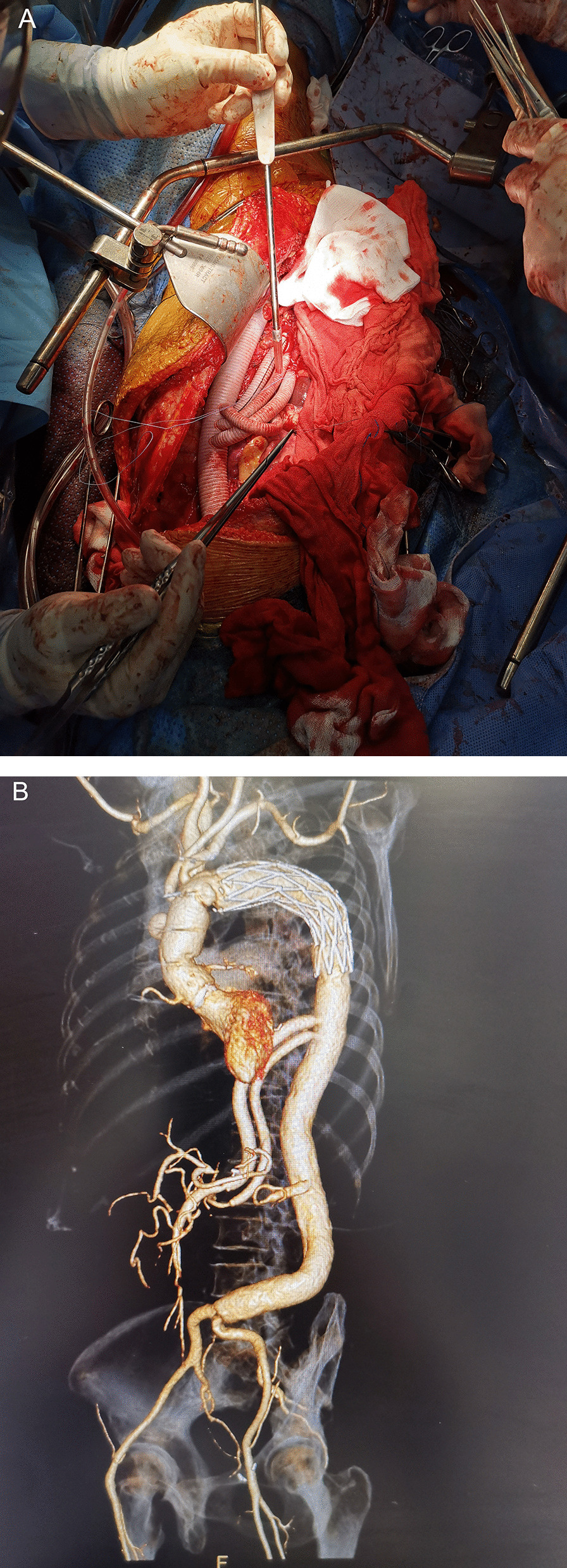
This patient successfully received the second-stage of thoracic–abdominal aortic replacement surgery (**A**). The postoperative CT (**B**) showed that the artificial blood vessel was normal in shape, the whole thoracic–abdominal aortic aneurysm was completely removed, and the revascularization of important organs was satisfactory

One month after the patient was discharged from our hospital, in order to avoid the residual thoracic–abdominal aortic aneurysm from rupturing, we performed the second-stage of surgery, that is, the patient underwent thoracoabdominal aortic replacement. A combined thoracic–abdominal incision was used, a double-lumen endotracheal tube was used during anesthesia, and a cerebrospinal fluid drainage tube was routinely inserted. The incision is made between the left scapula and the spine, bypassing the subscapular corner, along the posterolateral fifth intercostal space to the lower edge of the costal arch, and continuing to the rectus abdominis muscle and down to the iliac fossa. Enter the chest through the fifth intercostal space, traverse the costal arch, and cut off the diaphragm from the front to the posterior side at a distance of 3–4 cm from the chest wall along the edge of the diaphragm to reach the aortic hiatus. We used a total extraperitoneal approach to expose the abdominal aorta and its branch arteries, and performed thoracoabdominal aortic replacement surgery with a normal temperature (nasopharyngeal temperature 34–36℃) left heart bypass technique. After systemic heparinization, cardiopulmonary bypass was established through cannulation of the left inferior pulmonary vein, left femoral vein, and left femoral artery. Block the thoracic–abdominal aortic aneurysm at a distance of 2–3 cm from the distal end of the elephant trunk stent, transcribe the descending aorta, cut the aneurysm longitudinally, and suture the open intercostal artery. The proximal end of a suitable type of the 4-branch artificial blood vessel was anastomosed with the distal end of elephant trunk stent. After wrapping the lower intercostal artery into a vessel island, it was anastomosed end-to-side with the 4-branch artificial blood vessel to complete the reconstruction of the intercostal artery. Reduce the extracorporeal circulation flow to 1/2 of the full flow, block the external iliac artery cannulation, stop the circulation in the lower body, and complete the reconstruction of the abdominal aortic branch artery in turn. We usually use the superior mesenteric artery, celiac artery, right renal artery and left renal artery anastomosis sequence. After anastomosis of a blood vessel, immediately open a blood vessel to reduce ischemia time. Finally, the distal end of the abdominal aorta was anastomosed, and the thoracic–abdominal aorta replacement operation was completed (Fig. 3A). Fortunately, this female patient tolerated the operation well, without complications such as paraplegia, renal failure, or infection. The postoperative CT scan revealed that the thoracic–abdominal aortic aneurysm was completely treated with satisfactory results (Fig. 3B).

## Discussion

Patients with thoracic–abdominal aortic aneurysm usually have no symptoms until the lesion is severe enough to cause compression of the surrounding organs or dissection or rupture [1]. Therefore, the diagnosis of thoracic–abdominal aortic aneurysm is often accidentally discovered during imaging examinations due to other unrelated medical diseases. This female patient was asymptomatic, and a huge upper mediastinum mass was found on a conventional chest radiograph. In addition, once the patient develops symptoms, it often indicates that the aneurysms are about to rupture. The most common symptoms are chest, abdomen, and back pain. This pain is caused by aortic aneurysm compressing adjacent organs, or it may be caused by dissection or rupture. Clinically, these symptoms should be highly vigilant.

The diagnosis of thoracic–abdominal aortic aneurysm mainly relies on X-ray imaging [2]. Nowadays, aortic CT angiography (CTA) and 3-dimensional (3D) reconstruction have become the gold standard of preoperative imaging examinations, and aortic angiography is rarely needed anymore. CTA plays a vital role in formulating surgical strategies, especially when endovascular treatment is planned. Performing 3D reconstruction while observing the axial section will help to understand the anatomy of the aneurysm in detail. In this case, the lesion was first found on chest radiograph, prompting the physician to further check. CT examination confirms the diagnosis and provides detailed information for the design of the surgical plan.

For asymptomatic patients with total thoracic–abdominal aortic aneurysm, if the diameter of the aortic aneurysm exceeds 6 cm or the growth rate exceeds 1 cm per year, as long as there is no medical disease that is contraindicated, elective surgery should be performed. If the aortic aneurysm is less than 6 cm in diameter, but there are connective diseases, such as Marfan syndrome and Loeys–Dietz syndrome, surgery should also be performed. This female patient's aortic aneurysm has a maximum diameter of 72 mm, which has surgical indications and should be actively treated with surgery. In this case, we chose conventional open surgery instead of endovascular repair, mainly due to the following reasons: ① In recent years, with the great improvement in surgical technology, surgical instruments, artificial materials, etc., the mortality and paraplegia rate of total thoracic–abdominal aortic replacement surgery in China's major heart centers have been greatly reduced compared with the previous ones, and it has been reduced less than 10% [3]. ② Although endovascular repair can avoid cardiopulmonary bypass, left heart bypass, one-lung ventilation, reduce the time of visceral ischemia, and reduce blood loss. However, for thoracic–abdominal aortic aneurysms involving important branches of the abdominal aorta, endovascular repair alone cannot be performed well or the mid- and long-term effect is poor. ③ Due to the lack of prospective multi-center clinical randomized controlled experimental data, the treatment of total thoracic and abdominal aortic aneurysms is more based on personal experience or the level of surgical skills of surgeons or the level of endovascular repair skills of interventionists. ④ This female patient is a total thoracic–abdominal aortic aneurysm, involving a wide range of lesions, and moderate to severe aortic valve insufficiency. Based on our clinical experience, we prefer to surgical repair.

The entire thoracic–abdominal aneurysm involves start at the root of the aorta to the end at the bifurcation of the aorta-iliac artery. Surgery methods include staged total aortic replacement, elephant trunk staged total aortic replacement, or one-stage total aortic replacement [4]. One-stage total aortic replacement is the most complicated operation in aortic surgery, including the replacement and repair of the aortic root, ascending part, arch, descending thoracic aorta and abdominal aorta, involving the protection of all important organs of the human body, such as heart, brain, spinal cord, lungs, kidneys and liver. In addition, the operation time is long, the trauma is huge, and the postoperative complications and mortality are very high [5]. This patient is an elderly woman with obvious aortic calcification. In order to reduce the surgical risk, we finally chose the two-staged total aortic replacement with a stent elephant trunk, and the clinical results were satisfactory. This patient’s operation was very successful and her recovery was good. She is very grateful for everything we have done for her. She is willing and authorized to share her surgical treatment process to benefit more patients.

## Conclusions

In short, patients with total thoracic–abdominal aortic aneurysm may not have typical clinical symptoms, and clinicians need a comprehensive and detailed physical examination and targeted auxiliary examinations. Staged repair of total thoracic–abdominal aortic aneurysms is still a safe and effective treatment.

## Data Availability

Please contact authors for data requests.

## Abbreviations

CTA: Computed tomography angiography
CT: Computed tomography

## References

[1] Massimo CG, Presenti LF, Favi PP, Crisci C, Guadrónet EAC (1993). Simultaneous total aortic replacement from valve to bifurcation: experience with 21 cases. Ann Thorac Surg.

[2] LeMaire SA, Carter SA, Coselli JS (2006). The elephant trunk technique for staged repair of complex aneurysms of the entire thoracic aorta. Ann Thorac Surg.

[3] Cheng L, Huang F, Chang Q, Zhu J, Yu C, Liu Y (2010). Repair of extensive thoracoabdominal aortic aneurysm with a tetrafurcate graft: midterm results of 63 cases. Heart Surg Forum.

[4] Hu XP, Chang Q, Zhu JM, Yu CT, Liu ZG, Sun LZ (2006). One-stage total or subtotal aortic replacement. Ann Thorac Surg.

[5] Safi HJ, Miller CC, Estrera AL, Huynh TT, Rubenstein FS, Subramaniam MH (2001). Staged repair of extensive aortic aneurysms: morbidity and mortality in the elephant trunk technique. Circulation.

